# Could the re-emerging practice of wild boar hunting linked to the recent economic crisis lead to new outbreaks of trichinellosis in Lebanon?

**DOI:** 10.1051/parasite/2022011

**Published:** 2022-02-28

**Authors:** Georges Khalil, Pierre Marty, Karl Hage, Salma Sfeir, Jeanne El Hage, Tarek Bou Assi, Maria Rassam, Christelle Pomares, Elio Mikhael

**Affiliations:** 1 Medical Microbiology Department, Faculty of Medicine, Saint Joseph University P.O. Box 11-5076 – Riad El Solh 1107 2180 Beirut Lebanon; 2 Hôpital Saint Joseph des Sœurs de la Croix-Centre Médical Raymond et Aida Najjar P.O. Box 90-375 Bauchrieh, Dora-Metn Beirut Lebanon; 3 Service de Parasitologie-Mycologie, Université Côte d’Azur, Inserm U1065, Centre Hospitalier Universitaire de Nice 062020 Nice France; 4 Faculty of Medicine, Saint-Joseph University P.O. Box 11-5076 - Riad El Solh 1107 2180 Beirut Lebanon; 5 Animal Health Laboratory, Lebanese Agricultural Research Institute – LARI P.O. Box 90-1965, Fanar El Metn Lebanon; 6 Department of Laboratory Medicine, Hôpital Saint Joseph des Sœurs de la Croix-Centre Médical Raymond et Aida Najjar P.O. Box 90-375 Bauchrieh, Dora-Metn Beirut Lebanon

**Keywords:** Trichinellosis, Foodborne, Helminthiasis, Zoonosis, Wild boar

## Abstract

*Background*: Documented trichinellosis outbreaks in Lebanon date back to the late 19th century. The first published outbreaks were attributed to the consumption of wild boar meat, while those that followed incriminated pork. The practice of hunting wild boar is currently re-emerging in Lebanon given the recent economic crisis that has limited the purchase of livestock meat. *Results*: In Lebanon, at least 15 outbreaks of trichinellosis have been reported since 1870. We report an outbreak in January 2019, where five of the fifteen people present at a barbecue party were diagnosed with trichinellosis after wild boar meat consumption. Two subspecies of wild boar, *Sus scrofa libycus* and *Sus scrofa scrofa*, are commonly targeted by hunters. Hunters and consumers are sometimes unaware of the ineffectiveness of freezing meat and cooking over a wood fire to avoid trichinellosis. Unexpectedly, the National Center for Zoonosis Control receives every year 4 samples of wild boar meat, all free of *Trichinella sp.* larvae. *Conclusion*: Trichinellosis, a zoonosis typically unrecognized or undeclared, still represents a risk linked to the consumption of meat from wild animals, especially wild boar. Consumers, hunters, veterinarians, and butchers need to be further educated. Government regulation of wild boar hunting should be implemented to prevent further outbreaks.

## Abstract (fr)

*Contexte* : Les épidémies de trichinellose sont documentées au Liban depuis la fin du XIX^ème^ siècle. Les premiers foyers publiés étaient attribués à la consommation de viande de sanglier, tandis que ceux qui suivirent incriminaient le porc. La pratique de la chasse au sanglier est en train de réapparaître au Liban compte tenu de la récente crise économique qui a limité l’achat de viande de bétail. *Résultats* : Au Liban, au moins 15 foyers de trichinellose ont été signalés depuis 1870. Nous rapportons un foyer en janvier 2019, où cinq des quinze personnes présentes à une soirée barbecue ont reçu un diagnostic de trichinellose après consommation de viande de sanglier. Deux sous-espèces de sangliers, *Sus scrofa libycus* et *Sus scrofa scrofa*, sont couramment ciblées par les chasseurs. Les chasseurs et les consommateurs ignorent parfois l’inefficacité de la congélation de la viande et de la cuisson au feu de bois pour éviter la trichinellose. De manière inattendue, le Centre National de Contrôle des Zoonoses reçoit chaque année 4 échantillons de viande de sanglier, tous indemnes de larves de *Trichinella* spp. *Conclusion* : La trichinellose, zoonose généralement méconnue ou non déclarée, représente toujours un risque lié à la consommation de viande d’animaux sauvages, notamment de sanglier. Les consommateurs, les chasseurs, les vétérinaires et les bouchers doivent être mieux formés. Des réglementations gouvernementales sur la chasse au sanglier devraient être mises en œuvre pour prévenir de nouvelles épidémies.

## Introduction

Trichinellosis has been described many times in Lebanon, ranging from outbreaks to small series in the 20th century [2, 9, 11, 13–15, 17, 18, 20, 24, 27, 31]. Outbreaks were also reported in other Mediterranean countries, such as Greece, Italy, and Spain, during the past few years [1, 3, 7, 23, 26, 28].

In recent years, wild boar (*Sus scrofa*) hunting and wild boar meat consumption was the most described source of outbreaks. Bear and walrus meat consumption was also associated with confirmed trichinellosis in North America, Asia, and Europe [19, 25]. Wild boar meat consumption is associated with *Trichinella spiralis* infection as well as other species (*T. britovi*, *T. pseudospiralis*, and *T. papuae*) [25]. Undercooked meat and meat-derived products, as well as parasite load are among the most common described risk factors. Clinical signs and symptoms are mainly due to an intestinal phase consisting of diarrhea, abdominal pain, nausea and vomiting, and a larval invasive phase mainly causing fever, facial and/or orbital edema, cutaneous allergy, and myalgia [4]. Trichinellosis is a typical febrile helminthiasis.

During the past two decades, we have observed a drastic decrease in trichinellosis in Lebanon for many reasons, such as the prohibition by the state of an old practice of mixed butcheries (pork vs. other livestock). Moreover, pork meat consumption is limited to some areas in Lebanon as a result of confessional disparity, as well as the small number of pork farms due to more rigid health regulations recently imposed by the state. In contrast, the risk of trichinellosis has been increasing of late because wild boar hunting is becoming more popular in Lebanon. This hunting practice mainly takes place near the southern border of Lebanon, a zone that constitutes a true natural reserve sheltering wild boars, and that is yet to be regulated by the state. Other areas of Lebanon are also involved (Metn, Kesrouan, Chouf, and Bekaa valley). The recent financial crisis in Lebanon has caused basic needs shortages [30]. As a consequence, wild boar hunting has become a source of cheap protein-rich food.

The aim of this paper is to report a recent outbreak of trichinellosis due to wild boar meat consumption in Lebanon, in the context of renewed interest in wild boar hunting due to the recent economic crisis, and integrating it with historical epidemiologic data of trichinellosis in this country.

## Materials and methods

### Cases and definition of cases

Diagnostic criteria for trichinellosis were based on clinical symptoms and biological test results.

A confirmed case of trichinellosis was defined when a patient had consumed infested meat and had clinical signs and symptoms suggestive of trichinellosis, in addition to a positive muscle (deltoid) biopsy [4] or positive serology or seroconversion: considered negative if less than 11 NTU, or less than doubling value after 2 weeks [5].

A probable case of trichinellosis was defined when a patient had consumed infested meat followed by signs and symptoms such as intestinal phase symptoms (diarrhea, abdominal pain), as well as larval migration phase symptoms/signs (fever, myalgia, facial or periorbital edema, urticarial rash, and conjunctival or sub-nail hemorrhage). In addition, biological workup of the patient revealing any of the following biologic abnormalities suggestive of the disease resulted in a probable case: eosinophilia > 500 cells/μL (norm: 0–450 cells/μL), elevated creatinine phosphokinase (CPK, norm: 10–200 IU/L), or elevated lactate dehydrogenase (LDH, norm: 140–280 IU/L).

Complete blood count (CBC), CPK, and LDH testing was performed on all symptomatic individuals, while serological testing was done only for hospitalized patients.

Serum samples were tested using a commercial qualitative enzyme-linked immuno-sorbent assay (ELISA) for the detection of IgG class antibodies against *Trichinella* spp. (NovaTec Immun-diagnostica GmbH, Germany), according to the manufacturer’s recommendations. The manufacturer claims that the test has a diagnostic sensitivity and specificity of 100% and 94.81%, respectively and the testing kit was additionally validated in the laboratory (based on excretory/secretory *Trichinella* antigens) [5].

The index case allowed us to track and identify all other concomitant and/or consecutive cases by providing a list of people who consumed the wild boar meat.

### Literature review and inquiry with Lebanese wild boar hunters and the head of the Animal Health Laboratory, Lebanese Agricultural Research Institute (LARI)

In order to obtain information about previous trichinellosis outbreaks in Lebanon, general surveillance data were consulted on the official website of the Ministry of Public Health (MOPH). Furthermore, additional research was conducted on PubMed using the keywords “Lebanon” and “Trichinosis” or “Trichinellosis”. There were no restrictions on article time period.

In order to obtain information about the current status of trichinellosis in Lebanon, we interviewed the head of the Animal Health Laboratory at the Lebanese Agricultural Research Institute (LARI) and five private hunters from different regions of the country. LARI is a governmental institute under the supervision of the Ministry of Agriculture, responsible for all animal-derived products on the market. A particular research station located in Fanar, Lebanon (Mount Lebanon) has recently been established for animal health and zoonosis control.

## Results

### Case report in January 2019

The index case was a hunter hospitalized in January 2019 for low grade fever, eyelid and facial edema, myalgia, and diarrhea, with diffuse pruriginous maculo-papular rash (patient 1). His blood tests revealed hypereosinophilia, elevated CPK and LDH, and positive serology for trichinellosis. An investigation was then undertaken, upon which we discovered that fifteen people were present at a barbecue party and consumed hunted boar meat grilled over a wood fire. His hunting partner (patient 2) was simultaneously hospitalized for urticaria and fever in a different ward at the same hospital. His physician was notified about the high suspicion for trichinellosis in patient 1, and *Trichinella* serology testing confirmed the diagnosis. Three other probable cases were subsequently identified and treated as outpatients for similar but less intense symptoms (Table 1). They received symptomatic treatment for viral gastroenteritis. They were subjected to an oriented questionnaire and targeted blood tests were also performed that revealed hypereosinophilia and elevated CPK and LDH levels. The 10 remaining individuals were contacted for follow-up and serological testing, but refused to seek medical care since they were totally asymptomatic.

**Table 1:** Characteristics and symptoms described by patients.

Patient	Age – Sex	Fever onset post barbecue	Edema	Myalgia	Diarrhea onset	Skin manifestations	Eosinophilia (cells/mL)	Muscular enzymes level	*Trichinella* serology	Case category
1	32 – M	38 °C at day 5	Eyelid and facial	Yes	5 days	Diffuse maculo-papular rash with pruritis	8690	CPK 331	Positive (from 11 to >50 NTU)	Confirmed
								LDH 300		
2	35 – M	38.3 °C at day 3	Eyelid	Yes	4 days	Urticaria	7150	CPK 420	Positive (from 4 to 45 NTU)	Confirmed
								LDH 290		
3	28 – F	No	Facial	Yes	7 days	Diffuse pruritis	5500	CPK 300	NA	Probable
								LDH 280		
4	29 – M	No	Eyelid and facial	Yes	14 days	Generalized pruritis	6000	CPK 240	NA	Probable
								LDH 273		
5	30 – F	39 °C at day 7	Eyelid	Yes	3 days	Lower limb pruritis: maculo-papular rash	9070	CPK 350	NA	Probable
								LDH 160		

### Literature review and data collection in Lebanon

#### History of trichinellosis outbreaks in Lebanon from data of the MOPH

In Lebanon, trichinellosis is a reportable disease to the MOPH. Surveillance data that go back to 2002 on the MOPH website were checked for positive trichinellosis cases. There were only three reported cases from 01/01/2002 to 27/05/2020: one case in January 2016 in Beirut and our two cases reported in January 2019 (our confirmed cases). The trichinellosis cases reported to the MOPH and the cases from the literature in Lebanon are summarized in Table 2.

**Table 2:** Trichinellosis outbreaks in Lebanon.

Year	Location	Number of affected people	Source of infection	Notes	Reference
1871	Southern Lebanon	50	Wild boar meat	–	[20]
1881	Southern Lebanon	257	Wild boar meat	–	[31]
1939–1940	Beirut	500	Pork meat	–	[20]
1942	Beirut	2	Pork meat	–	[18]
1945	Mount Lebanon	36	Pork meat	–	[24]
1951	Northern Lebanon	40	Pork meat	–	[17]
1952	Beirut	6	Pork meat	–	[27]
1970	Mount Lebanon	37	Pork meat	–	[14]
1978–1979	Mount Lebanon, Beirut, Northern Lebanon, and Bekaa	89	Pork meat	–	[11]
1981	Southern Lebanon	>100 patients	Pork meat	–	[2]
1982	Southern Lebanon	>1000 cases (*estimated*)	Pork meat	–	[20]
1990–1991	East Beirut	200 (*estimated*)	Beef, sheep, and pork meat	This outbreak was not reported in the literature	Personal data from the corresponding author
1997	Southern Lebanon	44	Pork meat	200 patients were treated for *Trichinella*-like symptoms	[9]
2016	Beirut	1	Unknown	–	MOPH Surveillance data
2019	Beirut	2	Wild boar meat	Reported to the MOPH	Our case series

#### Data collection gathered from Lebanese wild boar hunters and the Head of the Animal Health Laboratory, LARI

There are two subspecies of wild boar in Lebanon. The first, *Sus scrofa libycus*, also known as the Anatolian boar, is mainly found along the southern border of the country. The second, the Central European boar (*Sus scrofa scrofa*), was imported in the late 20th century. The initial population of this subspecies, originally imported for leisure and hunting by private individuals, later on mated and became established in the northern regions of Lebanon.

In Lebanon, the state does not currently regulate wild boar hunting. The most recent legislation regarding this issue dates from 05 April 2017, when a law (Law 580/04) was passed to require physical, mental, and reading (or oral) examinations before obtaining a hunting license.

Based on our interview with the Head of Animal Health laboratory, *Trichinella* larvae detection has been done on pork and wild boar meat cuts. However, no data are available from before 2006. Unlike pork cuts, which are received from the private and public sectors, wild boar meat is tested on demand by private hunters. The samples were mainly received from the Metn and Aaley regions. Paraspinal muscles undergo trichinoscopy and pepsin and HCl digestion [16] for larva detection. An average of four wild boar samples per year are tested at this institute, with no positive tests for *Trichinella* larvae since 2006.

There is no official hunting season in Lebanon. Even though the regulations are scarce, there is a consensus between hunters. First, female wild boars are only hunted starting November until September of the following year, in order to allow time for the piglets to mature and be independent of their mother. Male wild boars are hunted independently of the season. Second, three hunting techniques are agreed upon and used: long distance sniping with thermal scopes, and short distance hunting with shotguns by baiting them either with food or hunting dogs. Third, hunters must aim for critical body parts (brain, heart, lungs, or spine) knowing that any non-fatal or non-debilitating shot would enrage the boar, leading to possible physical harm to the hunters or damage to the environment.

## Discussion

Although trichinellosis is a notifiable disease, the actual prevalence and incidence are unknown because the disease is difficult to identify due to a nonspecific picture or due to the absence of a pathognomonic clinical picture. Moreover, the disease could be self-limited without medical care seeking. Some outbreaks were not even published, such the one that occurred in East Beirut (Achrafieh) during the 1990–1991 war period. The estimated number of infected persons was around 200. At that time, the livestock was imported from Eastern Europe via Cyprus and the meat (beef, sheep, and pork) was delivered by the same butcher. *Trichinella* larvae were suspected to have been transmitted from one livestock to another by cross-contamination. Cross-contamination occurred because the same knives, cutting boards, and other utensils were used by the butcher to cut meat of different pieces of infected pork and those of sheep and beef meat. In the food traditions of the Lebanese people, eating raw sheep meat is common. Consuming rare sheep or beef meat is also usual. During this outbreak, the group that consumed the meat of pork or wild boar ate it well cooked. Data collected for 40 patients at this time by the corresponding author, for oral presentation, during his internship in the university hospital located in the epidemic area of trichinellosis, revealed that patients eating raw sheep meat were mainly those who subsequently developed the disease, knowing that raw sheep meat should be consumed within 6–8 h after preparation and delivery by the butcher, for a matter of taste. Only seven patients ate pork meat, and three ate rare beef meat.

There is a general misconception among hunters and consumers that freezing the meat kills larval worms. Studies, however, have shown that not all species of *Trichinella* are sensitive to cold temperatures. In fact, *T. spiralis* larval worms are neutralized by freezing the pork either 20 days at −15 °C or 3 days at −20 °C [10, 12, 29]. In contrast, other species found in wild boars, such as *T. britovi* and *T. nativa*, are able to resist cold temperatures [10, 12, 21, 29]. To kill the encysted larvae, internal meat temperatures must reach 62 °C or more, taking into consideration the variability in heat distribution and cooking techniques [6]. Using wood fire cooking, such as in our case has shown that heat distribution might not be optimal in the core of the meat to kill encysted larvae. In our reported outbreak, the most likely cause was that the meat was not successfully grilled, hence, a part was served rare. As a result, wild boar cuts should be handled with precaution when using wood fire cooking.

Furthermore, international veterinary societies recommend that hunters should refrain from killing ill-looking boars, as well as avoid leaving behind animal remains and waste after discarding unwanted meat, to avoid the spread of *Trichinella* in the wildlife [8, 22]. On the other hand, meat handlers (butchers and consumers) should follow certain hygiene rules (hand, utensil, and surface washing, with boiling water and diluted sodium hypochlorite solutions) [10, 12, 29].

As of 2020, Lebanon ranked as having the fourth highest inflation rate globally at 85.45%. This rapid hyperinflation has severely affected the purchasing power of the Lebanese people, especially in impoverished communities. As a result, prices of imports, particularly food and livestock have inflated by more than 441% since 2019. The most recent inflation rate stood at 144.1% during the month of October 2021. Meat prices have sky-rocketed and meat is no longer accessible to the lower- to average-income consumer. Consequently, other means of securing sufficient animal protein intake have been contemplated. One of these means is wild boar hunting for several reasons. First, pork meat consumption is not banned at the national level. Second, wild boars are abundant, as previously mentioned, and their hunting is unregulated. This creates a perfect recipe for gun-owning Lebanese who are looking for leisure or to provide food for their families.

In addition, the lack of veterinarian control increases the risk of unfortunate events. This is illustrated by the scarce amount of testing being done at the reference center for animal health control (LARI).

Our study was limited by the lack of further investigations to detect the species of *Trichinella* that was implicated in this case. It was not performed for financial reasons and futility regarding the management of the patients involved.

However, this is the first paper since the late 19th century that sheds light on trichinellosis outbreaks in Lebanon originating from wild boar meat consumption.

## Conclusion

Trichinellosis, a zoonosis typically unrecognized or undeclared, still represents a risk linked to the consumption of meat from wild animals, especially wild boar.

Given the looming risk of witnessing other trichinellosis outbreaks, the general population, and specifically veterinarians, hunters, and butchers, should be targeted by awareness campaigns regarding the disease and the risk of eating undercooked meat. They should also be instructed on how to properly prepare the meat in a risk-free manner. In addition, regulations should be implemented on this practice to avoid other outbreaks.
